# Comparison of Interventional Methods to Motivate and Change the Behavioural Stage of Smokers to Quit Smoking- A Hospital Based Randomised Controlled Trial

**DOI:** 10.31557/APJCP.2021.22.3.711

**Published:** 2021-03

**Authors:** Dipshikha Das, Ipseeta Menon, Ritu Gupta, Anubhav Sharma, Iram Ahsan, Asifa Ashraf

**Affiliations:** *I.T.S Centre for Dental Studies and Research, Delhi Meerut Road, Murad Nagar,Ghaziabad 201206, Uttar Pradesh, India. *

**Keywords:** Behavioural stage, intervention, motivate, smoking, smokers

## Abstract

**Objective:**

Addictions can be beaten if started off with a true motivation to quit it. Enhancing motivation is an important part of overall treatment for smoking cessation as it increases a smoker’s courage and enthusiasm to quit smoke. This study aimed to assess effectiveness of various interventional methods to motivate unmotivated smokers with a focus on changing behavioural stage of smokers to quit.

**Methodology:**

A single blinded trial was conducted at the outpatient department of ITS dental college and hospital among precontemplation stage smokers allocated into 4 groups and given interventional therapies like general counselling only(group 1), counselling and intra oral camera assessment (Group 2), counselling as well as carbon monoxide monitoring(Group 3)whereas fourth group(Group 4) given a combination therapy of all of them. A baseline evaluation of nicotine dependence and behavioural stage of the smoker was assessed. The patients were then evaluated on presence on their scheduled follow up visits done at interval of 2 weeks and 4 weeks. Descriptive statistics were addressed and the association was evaluated using Pearson chi square test. Any p value less than 0.05 was considered statistically significant.

**Results:**

Majority of smokers were males(88% ) and about 48.8% of them were highly dependent smokers . The change in precontemplation behavioural stage was assessed on basis of follow up visits after interventional therapy which was significantly higher in group 4 receiving combination therapy, followed by carbon monoxide therapy ,counselling and intra oral camera assessment and the least visits in behaviour counselling group (p<0.05).

**Conclusion:**

A combination of counselling and other motivational aids therapy is best way possible to help smokers focusing a change on the behavioural stage of the patient from precontemplation to preparation stage.

## Introduction

Tobacco is one of the most widely abused substance in the world. It is highly addictive. The Centres for Disease Control and Prevention estimates that tobacco causes 6 million deaths per year. According to the Global Adult Tobacco Survey (GATS) 2016-17, nearly 42.4% of men, 14.2% of women and 28.6% of all adults currently use tobacco in Smoked or smokeless form (GATS, 2016).

The Indian data suggest that the relative risk of developing oral cancer is 2.82 for smokers and 5.98 for chewers (Squier et al., 1984). Studies have also shown that almost 80% of oral cancers progress from precancerous lesions which mainly occurs due to consumption of various tobacco forms. Thus tobacco emerges as one of the leading and burning cause of preventable death in India.

Most of the people in India are aware of smoking or knows very well the health risks associated with it as we all know ‘Tobacco causes cancer’ is like a proverb to us which we encounter in every flip side of a cigarette packet, advertisements, hoardings, internet and what not but that does not make it any easier to kick this habit out. This is mainly because of the easy availability of the cigarette or tobacco products in almost each and every street of India. Individuals from different walks of life struggles with addiction to tobacco inspite of presence or absence of prevalence of these risk factors in their lives.

Addiction does not discriminate. Calling a cigarette or bidi abuse for yourself or for your loved ones is a difficult decision to make. It is not always easy admitting that you or your loved one is actually struggling with addiction. Addiction is an attachment so strong that the person experiences difficulty in avoiding the activity even when it causes harm (Roy et al., 2017). It is pretty normal that addiction can happen to anyone at any point of time. But the interesting fact is most of them don’t really like to admit that he/she is an addict starting from an occasional teen smoker to a whole packet per day user.

But this very powerful term addiction can be beaten, if started off with a true motivation to quit it. According to Mallin R, once the diagnosis of nicotine dependence is made, the next step is to assess the patient’s readiness to change (Mallin R et al., 2002). Enhancing motivation is an important part of overall treatment for smoking cessation as it can change behavioural stage of the smoker. The five-stage transtheoretical model for readiness to change can be applied to addictive behaviours such as smoking. The five stages in this model are precontemplation, contemplation, preparation, action, and maintenance. Patients often cycle through the stages of change several times before reaching stable abstinence (Prochaska et al., 1992). The utmost need of the hour is to incorporate counselling for tobacco cessation at the primary care level. It It is important to ensure that the population is well informed about availability and accessibility of tobacco dependence treatment services by encouraging them to make use of it (Goyal et al., 2020).

Thus, Quitting and staying smoke free can be a challenge but many smokers have actually done it. However, there has been no documented literature on the efficacy of tobacco cessation interventions based on the behavioural stage of the patient. This study aims at indulging various behavioural management therapies and treatment modalities among adult population attending the outpatient department of the institute in Ghaziabad that can actually help a smoker to succeed.

## Materials and Methods

*Study setting*

This study was conducted at the outpatient department of ITS dental college and hospital among the individuals reporting with a habit history of tobacco smoking. Trial recruitment took place between May 2019 and June 2019 and no important changes were made after the trial commencement. We obtained ethical approval from the Institutional Ethics Committee and all participants included in the study signed a detailed informed consent form that described the purpose of the study as well as their role and responsibilities prior to their participation. 

*Study design and randomisation*

A four arm, single blinded explanatory randomized controlled trial was conducted to assess the efficacy of various interventional therapies to motivate smokers to quit smoking by changing their smoking behaviour with the help of Transtheoretical model of behaviour change. Randomisation was conducted to a computer generated allocation sequence of 1:1:1:1 to the Behaviour counselling group (1), behaviour counselling and intra oral camera assessment (group 2), counselling as well as carbon monoxide monitoring (group 3) and a combination therapy of all of them (group 4). 

Researchers conducting research were blind to the group allocation, which occurred subsequently. Due to the nature of the study participants knew they were assigned to one of the groups. Neveretheless the participants were not informed about the motivational intervention method priorly. Post treatment assessments were carried out and follow ups were recorded at 2 weeks and 4 weeks interval. A phone call was also done in between the follow up visits to reinforce and remind the participants to continue the study.

*Participant recruitment*

A total of 242 participants(smokers) were included, out of which 80 smokers were in precontemplation stage who were recruited in the study further.

*Inclusion criteria –*

• Participants who were in precontemplation stage.

• Participants who have consumed tobacco (smoking) within a past period of 6 months

• Available for further follow up visits or should be able to attend counselling sessions to be a part of the intervention.

*Exclusion criteria –*

• Participants enrolled in other cessation programs or using any pharmacotherapy for cessation. 

• Pregnant/ breastfeeding mothers 

*Baseline assessment*

Baseline measures included patient demographics, smoking history, withdrawal symptoms with past quit attempts, triggers for smoking, current withdrawal symptoms, motivation to quit, history of smoking behavior changes, and measures of addiction to nicotine (using a modified Fagerstorm nicotine dependence scale (Heatherton et al., 1991).

Identification of the behavioural stage of the smoker - Nicotine that is present in tobacco is highly addictive so a person goes through various behavioural stages in an attempt to actually quit tobacco according to the transtheoretical model given by Diclemmente and Prochaska, (1992). 

The behavioural stage of the smoker is assessed by asking a simple question to them that ‘ Do you smoke?’, If the patient says a no means he is in the precontemplation stage that is the patient does not believe that smoking is a problem or refuses to consider smoking cessation.

*Interventions- The course of the intervention was 1 month duration*

• GROUP 1 (Behaviour counselling )– Participants randomized to this group were given an individual behavioural counselling where they were informed about the various health risks associated with smoking. Specific counselling messages were solely based on the individual’s stage of readiness to quit smoking and was based on the “5 R’s” (i.e., relevant risks of smoking, rewards of quitting, roadblocks to cessation, repetition at each visit).

•GROUP 2 (Behaviour counselling and intra oral camera assessment)- Intra oral camera assessment along with a behaviour counselling was used which helped the participants to have a view of their tobacco devastated mouth with the purpose of diagnosing epithelial precursor lesions (leukoplakia, erythroplakia and actinic cheilitis)and precancerous conditions (lichen planus) which are common oral lesions found in tobacco users.

• GROUP 3(Behaviour counselling and carbon monoxide monitoring)- Participants were given a behaviour counselling along with a Smokerlyzer R9(Bedfont Scientific Ltd.) carbon monoxide(CO) monitor test which provides an unbiased indication of smoking habit. The main focus was to assess the CO levels in their lungs and discuss the adverse effects of this gas on their body. With the aim of maintaining their CO reading below 10 ppm (a threshold representing very light smoking) they were asked to quit smoking. 

• GROUP 4 (Behaviour counselling, intra oral camera assessment and carbon monoxide monitoring)- Participants randomized to this group were given a triad therapy of individual behavioural counselling, intraoral camera assessment and carbon monoxide monitoring at their baseline visits.

*• Follow up visit (Contemplation stage/Preparation stage )*

The sequential follow up visits were recorded at 2 weeks and 4 weeks interval time . For those wanting to quit, counsellors used guidelines that included changing environmental triggers, preparing for obstacles, self-rewarding and setting a quit date. Counsellors were trained to maintain an “advice-oriented” style of counselling during quit planning. Subsequent follow up sessions reviewed progress with the behavioural stage of the smokers to make a quit plan.

Apart from these, each group was given the subjected intervention which was given in the baseline.

a) First follow up visit (Contemplation stage) – The presence of the smoker on the return first follow up visit reveals that the patient now agrees that smoking is a problem and would like to consider quitting, revealing that the patient has now entered the contemplation stage of behaviour to quit smoking

Intervention- Reinforcement of the interventional methods in all the four groups. Interventions also included providing further education about the effects of smoking and encouraging the patient to consider the positive aspects of not smoking, such as improved health, a more positive self image, and economic savings. 

Subjects were now asked to maintain a tobacco use journal in their day to day life where they could keep a track record of their nicotine craving history and were asked to bring the same in their second follow up visit.

b) Second follow up (Preparation Stage)- His/her second follow up visit will reveal that Once the patient agrees that the benefits of not smoking outweigh the pleasure derived from smoking and has decided to quit, he or she has entered the preparation stage.

Intervention - After assessing their track record dairy, subjects were now advised to keep themselves busy, remove tobacco products from his/her surroundings, listen to music and to exercise. All these motivational messages were repeated at every follow up visit and they were now asked to themselves to set a quit date to quit smoking.

*Statstical Analysis*

The procured data were entered into the Microsoft Excel (Microsoft Corporation) and were subjected to the statistical analysis using SPSS software version 19.0 (SPSS Inc., Chicago, IL, USA). Descriptive statistics were addressed and the association was evaluated using Pearson chi square test. Any p value less than 0.05 was considered statistically significant.

## Results

*Demographic characteristics*

Table1 depicts the sociodemographic characteristics of the study participants. The present study was conducted among 18 to 57 years precontemplation adult smokers with a mean age of 41.3 ± 14.97 years. 88.8% male smokers and 11.2 female smokers participated in the study. Among 80 participants, 45% of them belonged to the semi urban locality, 35% from the rural and 20% from the urban locality. 

A majority of 43.8% of the smokers were cigarette users which was followed by 36.2% beedi smokers. The analysis of FTND scale assessment showed that a majority of 48.8% smokers were highly nicotine dependent, followed by 33.8% moderately dependent and 17.5% minimally/low nicotine dependent. High prevalence of oromucosal lesions were also found among smoker. Among these, 27.5% of them had smokers palate, 20% had leukoplakia and 6.2% had oral submucous fibrosis

Table 2 depicts the number of first and second follow up visits after the motivational intervention to change the behavioural stage of the precontemplation smokers. A majority 37% of the first follow up visits and 40% second follow up visits were observed by the counsellor among the participants receiving a triad therapy of behaviour counselling, intraoral camera assessment and CO monitor whereas the least number of 1st (21.7%) and 2^nd^ (12%) follow up visits was observed among participants receiving only behaviour counselling. Among group 2 and 3 participants, 13% and 28.3% came for the first follow up visits respectively. Similarly, 24% of the Group 2 and 3 participants came for the second follow up visits to the counsellor. 

A statistically significant difference was found among the all the study groups based on their first follow up visit.(p≤0.05%) whereas there was no significant difference found among the study groups in their second follow up visits. 

**Table 1:** Distribution of Study Subjects According to Sociodemographic Characteristics

Variables	Number (N)	Percentage
Age group (in years)		
18-27 years	20	25.0
28-37 years	13	16.2
38-47 years	19	23.8
48-57 years	28	35.5
Gender		
Males	71	88.8
Females	9	11.2
Geographic Location		
Urban	16	20.0
Semiurban	36	45.0
Rural	28	35.0
Socioeconomic status		
Lower	4	5.0
Upper Lower	18	22.5
Lower middle	48	60.0
Upper middle	8	10.0
Upper	2	2.5
Type of smoking form of tobacco used		
Cigarette	35	43.8
Beedi	29	36.2
Hookah	5	6.2
Any other/combined users	11	13.8
Severity of nicotine dependence (as per Fagestrom nicotine dependence scale)		
Highly dependent	39	48.8
Moderately dependent	27	33.8
Minimally dependent	14	17.5
Oromucosal lesions		
No lesions	30	37.5
Smokers palate	22	27.5
Leukoplakia	16	20.0
Osmf	5	6.2
Others	7	8.8

**Table 2:** Distribution of Study Groups Based on Their Follow up Visits

		Group 1(%)	Group 2(%)	Group 3(%)	Group 4(%)	Pearson chi-squared	P-value
Follow up 1 visit	No	29.4	41.2	20.6	8.8	13.299	0.004
	Yes	21.7	13.0	28.3	37.0		
Follow up 2 visits	No	30.9	25.5	25.5	18.2	5.76	0.124
	Yes	12.0	24.0	24.0	40.0		

**Figure 1.**
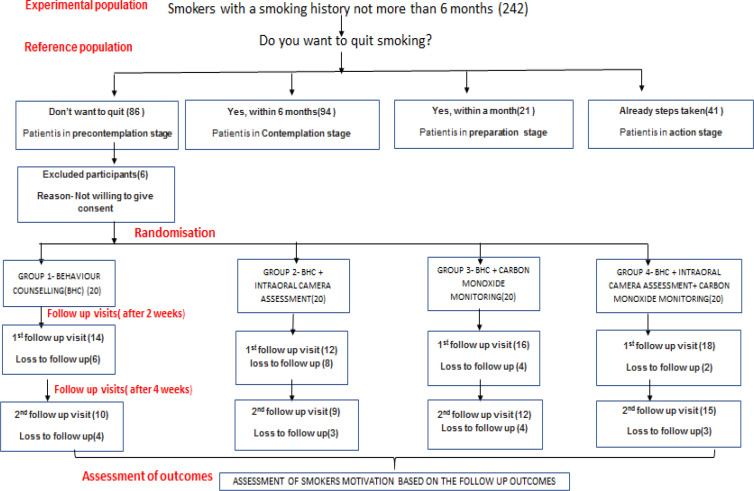
Study Design of a Randomised Controlled Trial

## Discussion

Since decades, smoking is known to be a risk factor and a major preventable cause of morbidity and mortality rates among people (Menon et al, 2019). A dentist need to develop techniques to assist patients who will benefit from a behaviour change. Traditional advice and patient education does not work with all patients. Understanding the stages through which patients pass during the process of quitting smoke enables a counsellor to tailor interventions individually. Therefore this study outlines a motivational approach that can increase the likelihood of successful smoking cessation by assessing the behavioural stage of the smokers at a dental hospital visit of the smokers.

In the present study, the prevalence rate of smoking was higher among the males (88%) followed by 11.2% females which is similar to studies done by Rani et al., (2003) and Kadtane et al., (2014). The reason might be due to the low utilization of smoking among females as in a country like India, the social acceptance of females consuming smoking form of tobacco is low. While this is contrary to a review done by Menon et al., (2012) where tobacco companies are seen targeting women and girls with visions of glamour, independence to lead a beautiful life resulting in significant rise of women smokers in developing countries.

According to a study (Hiscock et al., 2012), smoking prevalence is always higher among the low socioeconomic and disadvantaged groups of the society facing higher exposure to tobacco’s harms. The analysis of the present study also showed that the major dominance of the smoked tobacco users were from lower middle(60%) and upper lower socioeconomic class (22.5%), which might be due to lack of motivation and knowledge about ill effects of tobacco along with stressful workload, reduced social support for quitting, psychological differences such as lack of self-efficacy and tobacco industry marketing.

In the present study, the predominance of cigarette(43.8%) and bidi users(36.2%) were higher among the smokers due to the easy availability as well as affordable price of these commodities among the local markets of Muradnagar. 

The present study showed that majority of the participants were highly dependent smokers(48.8%) which urged an attention to the necessities for building up a suitable instructive, preventive and treatment measure combined with successful reconnaissance for tobacco end among the population in Goyal et al., (2019).

Oromucosal lesions like leukoplakia (20%) and smokers palate (27.5%) were majorly seen in this study which is similar to study done by Ikeda et al., (1995) and Machigo et al., (1995) who reported leukoplakia predominantly common in smokers. This might be due to dysplastic changes that occurs in the cells of the oral mucosa among the smokers due to the toxic contents present in tobacco. Therefore, the oral mucosa should be examined carefully, especially in smokers, even if the patients did not attend with the complaint of oral lesions caused by tobacco as it is a good way to initiate tobacco cessation (Ahmadi et al., 2013). Dentists should be aware of the adverse effects of smoking which can lead to oral pathologic lesions and encourage smokers to quit. Smoking may be a predisposing factor in the development of gingival problems as well. 

The present research has enabled a comprehensive comparative evaluation of four most viable techniques of motivating precontemplation smokers to quit on an outpatient setup. The sole reason for opting smokers at the precontemplation stage is to empathetically engage patients in contemplating change of his behaviour. During this stage, patients appear argumentative, hopeless or in “denial,” and the natural tendency is for the counsellor is to try to “convince” them by using various motivation methods.

It was observed that the group receiving a triad therapy of behaviour counselling + intraoral camera + carbon monoxide monitoring had greater follow up rates leading to a change in their behaviour stage to quit tobacco from precontemplation to comtemplation (37%) and then finally to preparation stage (40%). This finding might be due to the reason that a triad combination of motivational counselling is practically more applicable approach than any of the other combination therapies alone since the smokers here could visually see their oral lesions, analyse the CO levels instantly as well as get the right advice from his counsellor which made him easier to believe their dentist words influencing their behaviour. However no statistically significant difference was found among comparison of the study groups in the second follow up visits. 

Group 3 (behaviour counselling+ CO monitor) first and second follow up rates were comparatively higher (28.3% and 24% respectively ) than group 1 and 2. CO in breath is a form biofeedback of the smokers’ own body. Psychologically, it helps in a way that smokers feel their own body is being harmed by the gas and they can control the damage by stopping smoking. This is comparable to study done by Sejourne et al., (2010) and Beard et al., (2012), where researchers found that when advice from counsellor was coupled with using CO analyzer results as biofeedback, the smokers were more influenced and more motivated to quit.

The follow up rates were minimum in the group 1 receiving only behaviour counselling. This is comparable to a study in British Medical Journal which compared three different antismoking interventions using a controlled trial. The group which received antismoking advice and demonstration of exhaled CO had the highest smoking cessation rate, outperforming control, group which received only advice and group which received advice plus offer of further help from a health visitor (Jamrozik et al., 1984).

Overall , the follow up rates were low in all the 4 groups because of the deeply ingrained cultural habits particularly amongst those from rural areas (Murthy et al., 2010). This has been reflected in studies conducted in India, Nepal, Pakistan and United Kingdom south Asian population where cultural and social factors have led to the decline in promotion of effective smoking cessation practices (Kakde et al., 2012).

This study is first of its kind among the population of Muradnagar district since no research is available that uses the Stages of Change model in a hospital setting using motivational interventions. 

Apart from the strengths of the study, a source of bias that might have crept in that is over dependence upon self-reported cessation status as some of the participants might have misquoted their smoking status (smokers who were identified as quitters) and, moreover, in this study, only the shortterm intervention effects (2 weeks and 4 weeks) were assessed, but longterm assessments would be needed to assess the endurance of interventional effects.

In conclusion, a combination of counselling and other motivational aids therapy is the best way possible to help smokers to quit smoking by focusing a change on the behavioural stage of the patient from precontemplation to preparation stage. A basic assessment of a patient’s readiness to adapt a change, appreciating barriers to change and providing a helping hand to them to anticipate relapse can eventually improve the satisfaction of patients and lower the frustration of the physician as well during the process.

*Recommendations*

1. Dentists can enlist the help of other health care professionals (e.g., nutritionists, nurses, mental health personnel) to reinforce the message that a change in behaviour is needed and to provide additional education and skill information to the patient. 

2. Referral can also reduce some patient care burden for the dentist. 

3. Dentists should document the content and outcome of patient conversations, including specific tasks and plans for follow-up.

*Key message*

Tobacco is and always be one of the biggest public health concern for us.So as a healthcare professional, it is our duty to do the best to eradicate this from the very grassroot level.

## Author Contribution Statement

1. Dr. Dipshikha Das - Concept Design, Data Acquisition, Literature Search, Data Analysis, Statistical Analysis and Manuscript Preparation.

2. Dr. Ipseeta Menon- Concept Design, Literature Search, Manuscript Preparation, Manuscript Editing and Manuscript Review.

3. Dr. Ritu Gupta- Concept Design, Literature Search, Data Analysis, Statistical Analysis, Manuscript Preparation, Manuscript Editing and Manuscript Review.

4. Dr.Anubhav Sharma - Concept Design, Literature Search, Manuscript Preparation, Manuscript Editing and Manuscript Review.

5. Dr. Iram Ahsan - Manuscript Preparation and Manuscript Editing.

6. Dr. Asifa Ashraf - Manuscript Preparation and Manuscript Editing
